# ﻿Two new genera (*Vittiblatta* gen. nov. and *Planiblatta* gen. nov.) of Blattinae (Blattodea, Blattidae) from Southwest China and the discovery of chirally dimorphic male genitalia in *Vittiblattapunctata* sp. nov.

**DOI:** 10.3897/zookeys.1187.113403

**Published:** 2023-12-28

**Authors:** Xin-Xing Luo, Wen-Bo Deng, Yan-Li Che, Zong-Qing Wang

**Affiliations:** 1 College of Plant Protection, Southwest University, Beibei, Chongqing 400715, China Southwest University Chongqing China; 2 Key Laboratory of Agricultural Biosafety and Green Production of Upper Yangtze River (Ministry of Education), Southwest University, Chongqing 400715, China Southwest University Chongqing China

**Keywords:** Chiral dimorphism, male genitalia, new species

## Abstract

This study examines Blattinae samples from Southwest China collected in recent years. Based on morphological characters, we establish two genera, *Vittiblatta***gen. nov.** and *Planiblatta***gen. nov.**, and describe four new species, *Vittiblattapunctata* Luo & Wang, **sp. nov.**, *Vittiblattaferruginea* Luo & Wang, **sp. nov.**, *Vittiblattaundulata* Luo & Wang, **sp. nov.**, and *Planiblattacrassispina* Luo & Wang, **sp. nov.** These two new genera resemble *Periplaneta* s.s., but are easily distinguished from it and other genera of Blattinae by morphological characters (genital sclerite L4C). Our results indicate that sclerites L4C and R1G of male genitalia might be important in species delimitation of Blattinae. In addition, chiral dimorphism is found in male genitalia of *Vittiblattapunctata* sp. nov.

## ﻿Introduction

Blattinae Latreille, 1810, the nominotypical subfamily of Blattidae Latreille, 1810, presently includes 25 genera and about 262 species worldwide (Beccaloni 2023). They are mainly distributed in the Oriental, Australian, and Afrotropical realms. In recent years, molecular studies have revealed that Blattinae is non-monophyletic and the subfamily has been revised accordingly (Wang et al. 2017; Evangelista et al. 2018; Liao et al. 2021; Djernæs and Murienne 2022; Deng et al. 2023), resulting in the rediagnosis of Blattinae (Deng et al. 2023).

*Periplaneta* Burmeister, 1838 (*sensu lato*) is the most species-rich genus of Blattinae in China. This genus has been shown to be largely polyphyletic in recent studies (Legendre et al. 2015; Bourguignon et al. 2018; Liao et al. 2021; Djernæs and Murienne 2022; Li et al. 2022; Deng et al. 2023; Malem et al. 2023), and it should be divided into at least four separate taxa (Deng et al. 2023). This genus and most related genera were distinguished by a few external morphological characters (e.g. Asahina 1980; Bohn 1985), but with the increasing number of species, genital features should be given more consideration. Lucañas (2023) started to revise *Periplaneta* and established two genera, *Hobbitoblatta* and *Nazgultaure*, based on male genitalia. Luo et al. (2023) then proposed synapomorphies of *Periplaneta* s.s. by comparative morphology, based on the type species, *P.americana* (Linnaeus, 1758), and two species that were previously placed under *Shelfordella* Adelung, 1910.

In this study, we examine Blattinae samples from Southwest China and find four new species by morpholo-anatomic characters. The external morphology of these four species is similar to *Periplaneta* s.s., but they can be clearly distinguished by male genitalia. We also compare their morphology with that of other genera of Blattinae and conclude that these four species should be grouped into two new genera, which we establish here. In addition, we found chirally dimorphic male genitalia in one of the new species.

## ﻿Materials and methods

### ﻿Specimen source and treatment

Blattinae specimens from Southwest China were stored in anhydrous ethanol at −20 °C. Male and female genitalia were placed in 10% NaOH at 70 °C for 10 min to dissolve soft tissue, they were observed under a CNOPTEC SZ780 stereomicroscope, then stored in glycerol. Images were taken with a Canon M5 camera with a Laowa 65 mm F2.8 CA-Dreamer Macro 2× macro lens or a Leica M205A stereomicroscope with a Leica DFC 550 camera, and edited with Adobe Photoshop CC2019. All materials examined are deposited in the College of Plant Protection, Southwest University, Chongqing, China (**SWU**).

### ﻿Morphological terminology

In this paper, the terminology mainly follows Roth (2003), Li et al. (2018) (veins), Klass (1997) (male genitalia), and McKittrick (1964) (female genitalia). The abbreviations used are as follows: cubitus (**Cu**), cubitus anterior (**CuA**), cubitus posterior (**CuP**), media (**M**), postcubitus (**Pcu**), radius (**R**), radius anterior (**RA**), radius posterior, (**RP**), subcostal posterior (**ScP**), vannal veins (**V**); sclerites of left phallomere (**L1**, **L2**, **L3**, **L4C**, **L4D**, **L4E**, **L4G**), sclerites of right phallomere (**R1G**, **R1H**, **R1F**, **R2**, **R3**); tergum X (**TX**), first valve (**v.I.**), first valvifer (**vlf.I**), second valve (**v.II**), posterior lobes of valvifer II (**p.l.**), laterosternite IX (**ltst.IX**), anterior arch (**a.a.**), spermathecal plate (**sp.pl.**), spermathecal opening (**sp.o.**), basivalvulae (**bsv.**), laterosternal shelf (**ltst.sh.**).

## ﻿Taxonomy

### ﻿ Vittiblatta

Taxon classificationAnimaliaBlattodeaBlattidae

Luo & Wang gen. nov.

DE390201-BBD4-5920-BEC9-F9612EE564CC

https://zoobank.org/89FF89FE-72B0-4D24-A2E4-A63B67CB2AFD

#### Type species.

*Vittiblattapunctata* Luo & Wang, sp. nov.

#### Diagnosis.

Some typical characteristics indicate that *Vittiblatta* gen. nov. belongs to the subfamily Blattinae (front femur of type A_2_, tarsi long and slender, cerci long and distinctly segmented, subgenital plate symmetrical). The new genus differs from the other genera of Blattinae as follows:

1. This sexually dimorphic genus can be distinguished from sexually monomorphic genera. **Apterous**: *Apterisca* Princis, 1963; *Brinckella* Princis, 1963; *Macrostylopyga* Anisyutkin, Anichkin & Thinh, 2013; *Miostylopyga* Princis, 1966. **Micropterous**: *Afrostylopyga* Anisyutkin, 2014; *Henicotyle* Rehn & Hebard, 1927; *Neostylopyga* Shelford, 1911. **Macropterous**: *Dorylaea* Stål, 1877; *Eroblatta* Shelford, 1910a; *Hobbitoblatta* Lucañas, 2023; *Homalosilpha* Stål, 1874; *Mimosilpha* Bey-Bienko, 1957; *Nazgultaure* Lucañas, 2023; *Thyrsocera* Burmeister, 1838.
2. This genus (tegmina and wings of male developed, tegmina of female only reaching the first tergite of abdomen) can be distinguished from the genera in which the female are apterous (*Archiblatta* Snellen van Vollenhoven, 1862, *Catara* Walker, 1868, *Deropeltis* Burmeister, 1838) and micropterous (*Pseudoderopeltis* Krauss, 1890; *Blatta* Linnaeus, 1758; *Planiblatta* Luo & Wang, gen. nov.).
3. Hind metatarsus of this genus is longer than or equal to the remaining tarsal segments combined and therefore different from *Eumethana* Princis, 1951 and *Scabinopsis* Bey-Bienko, 1969.
4. This genus has visible tergal gland and can be distinguished from *Cartoblatta* Shelford, 1910b, *Periplaneta* s.s., and *Blatta*.
5. In male genitalia, sclerites L4C and R1G can be used for distinguishing genera in Blattinae. L4C of this new genus is thin, ribbon-like and its basal part has densely spiny process; R1G of this genus has a curved spine. These characters are readily different from that of *Archiblatta*, *Blatta*, *Bundoksia* Lucañas, 2021, *Catara*, *Hobbitoblatta*, *Homalosilpha*, *Mimosilpha*, *Nazgultaure*, and *Protagonista* Shelford, 1908 (Wang et al. 2016; Liao et al. 2021; Lucañas 2021; Li et al. 2022; Deng et al. 2023; Lucañas 2023; Luo et al. 2023). These two sclerites are similar between this genus and *Periplaneta* s.s., but the distal part of L4C of *Periplaneta* s.s. is expanded and the hind margin of L4C is nearly truncated.

#### Generic description.

Sexual dimorphism. **Male.** Interocular space wider than interocellar space, shorter than the distance between antennal sockets. Antennae longer than the body. Pronotum subelliptical. Tegmina and wings well developed, surpassing the tip of abdomen. Front femur of type A_2_; pulvilli present on 1–4 or 2–4 tarsal segments, claws symmetrical and unspecialized, arolium slightly smaller than other genera. The posterior-lateral angles of metanotum without or with small projections. First tergite of male abdomen with visible gland. Posterolateral corners of abdominal tergites V–VII not produced. The hind margin of supra-anal plate slightly concave. L1 of genitalia weakly sclerotized with pubescence; L3 unciform and the distal part bifurcated; L4C thin ribbon-like, with densely spiny process near basal inner margin. The basal part of R1H flat, inner margin with one or two small spines; the distal part of R1G with a curved spine inward. **Female.** Tegmina and wings reduced. Tegmina squamiform, only reaching the first tergite of abdmen; lateral margins of tegmina beveled, the outer corner rounded. Hind wings small and lobe-like. Pulvilli present on 1–4 or 2–4 tarsal segments, claws symmetrical and unspecialized, arolium small. Spermatheca with two branches, the leading duct short, the branching duct relatively long, and the end capsule rod-shaped.

#### Etymology.

The generic epithet is from two Latin words “*vitta*” and “*blatta*”, meaning that L4C is thin and ribbon-like. The gender of *Vittiblatta* is feminine.

#### Distribution.

China (Sichuan, Yunnan).

### ﻿Key to species of *Vittiblatta* Luo & Wang, gen. nov. (males)

**Table:** 

1	Pronotum with punctures	***V.punctata* Luo & Wang, sp. nov.**
–	Pronotum smooth, without punctures	**2**
2	The hind margin of subgenital plate convex	***V.ferruginea* Luo & Wang, sp. nov.**
–	The hind margin of subgenital plate wave-like	***V.undulata* Luo & Wang, sp. nov.**

### ﻿ Vittiblatta punctata

Taxon classificationAnimaliaBlattodeaBlattidae

Luo & Wang sp. nov.

BEEBD661-6E0A-57F5-B5B9-0AE5A09C129D

https://zoobank.org/337CEA3C-952C-4AEC-A463-15C46682CEB0

**Figure 1.**
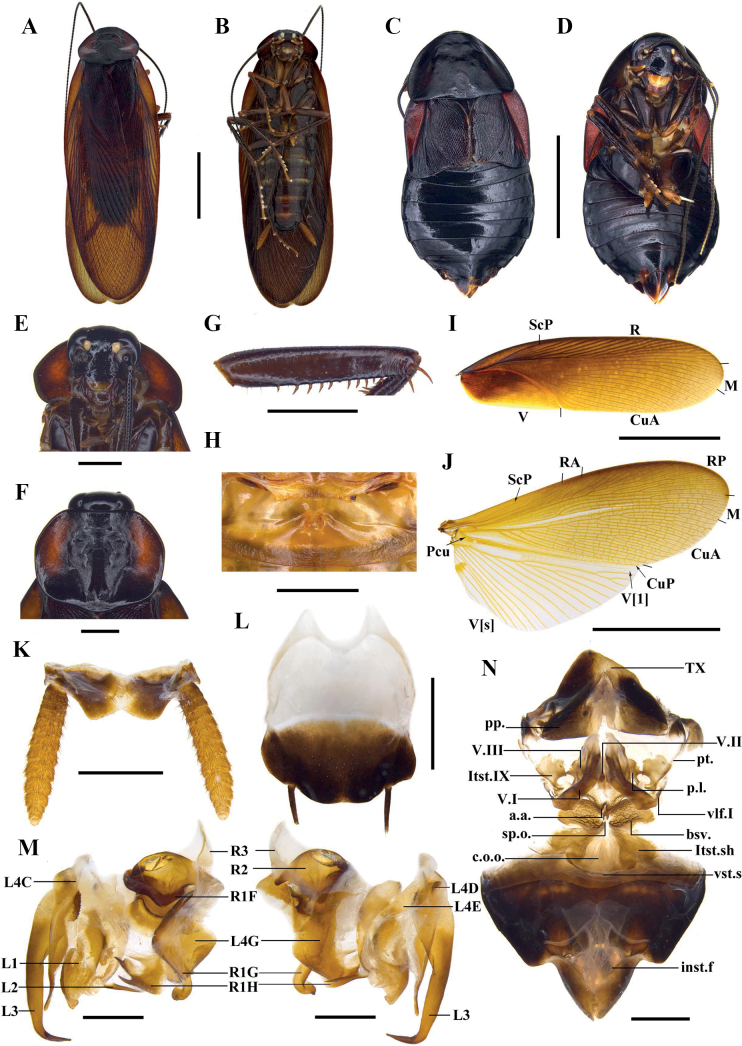
*Vittiblattapunctata* Luo & Wang, sp. nov. **A, B, E–M** male holotype **C, D, N** female paratypes **A, C** habitus, dorsal view **B, D** habitus, ventral view **E** head **F** pronotum **G** front femur **H** hind margin of metanotum and tergal gland **I** tegmen **J** hind wing **K** supra-anal plate, ventral view **L** subgenital plate, dorsal view **M** male genitalia, dorsal (left) and ventral view (right) **N** female genitalia, dorsal view. Scale bars: 10.0 mm (**A–D, I, J**); 2.0 mm (**E–H, K, L, N**); 1.0 mm (**M**).

**Figure 2.**
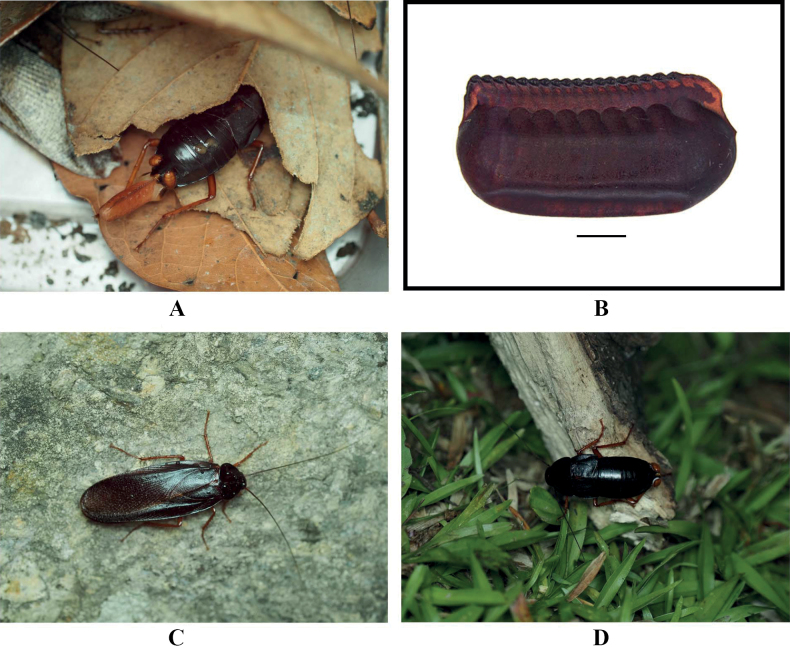
*Vittiblattapunctata* Luo & Wang, sp. nov. **A** ootheca-carrying female **B** ootheca **C** male on rocks **D** female in the grass. Scale bars: 2.0 mm (**B**). **A, C, D** photographed by Xinran Li.

**Figure 3.**
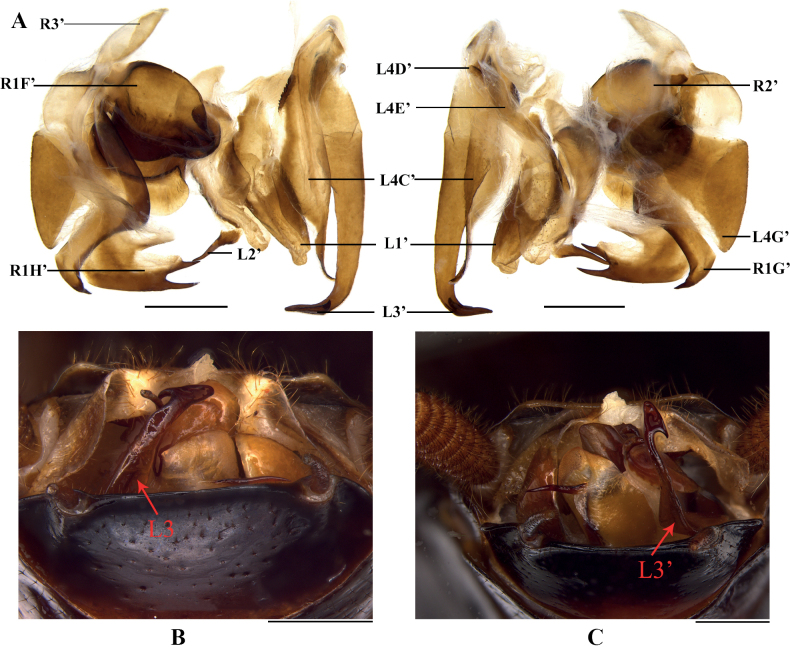
Chiral dimorphism in male genitalia of *Vittiblattapunctata* Luo & Wang, sp. nov. **A–C** male paratypes, the samples from Mt Ailaoshan **A** mirrored genitalia, dorsal and ventral views **B** normal genitalia, ventro-caudal view (L3 on the left) **C** mirrored genitalia, ventro-caudal view (L3 on the right). Scale bars: 1.0 mm.

Figs 1 , 2 , 3 , 7 (in part)

#### Type materials.

***Holotype***: China • ♂; Sichuan, Miyi County, Panzhihua City; 20.VII.2021; Lu Qiu leg.; SWU-B-BL-083301. ***Paratypes***: China • 1♂; Sichuan, Mt Lushan, Xichang City, Liangshan Autonomous Prefecture; 21.VII.2022; Bianlun Li & Lin Guo leg.; SWU-B-BL-083302 • 1♂; Sichuan, Mt Lushan, Xichang City, Liangshan Autonomous Prefecture; 1800 m alt.; 30.VI.2015; Chao Zhou leg.; SWU-B-BL-083303 • 1♀; Sichuan, Mt Lushan, Xichang City, Liangshan Autonomous Prefecture; 21.VII.2022; Wei Han & Xinxing Luo leg.; SWU-B-BL-083304 • 2♂♂; Sichuan, Mt Daheishan, Panzhihua City; 20–21.V.2011; Keliang Wu leg.; SWU-B-BL-083305 to 083306 • 5♂♂; Yunnan, Mt Ailaoshan, Xinping County; 1988 m alt.; 24.V.2018; Lu Qiu, Wenbo Deng & Zhiwei Dong leg.; SWU-B-BL-083307 to 083311 • 1♂, 2♀; Yunnan, Mt Ailaoshan, Xinping County; 1988 m alt.; 11–13.V.2016; Lu Qiu & Zhiwei Qiu leg.; SWU-B-BL-083312 to 083314 • 4♀♀; Yunnan, Xishan Scenic Area, Kunming City; 2240 m alt.; 27.VI.2021; Jiawei Zhang & Jinlin Liu leg.; SWU-B-BL-083315 to 083318 • 1♂; Yunnan, Wenquan Street, Kunming City; 1900 m alt.; 3–4.VI.1974; Yao Zhou & Feng Yuan leg.; SWU-B-BL-083319 • 1♂; Qiongzhu Temple, Kunming City; 2166 m alt.; 14.VI.1980; collector unknown; SWU-B-BL-083320 • 2♂♂; Yunnan, Mengxima Town, Yingjiang County, Dehong Autonomous Prefecture; 1470 m alt.; 9.VI.2008; Weiwei Zhang leg.; SWU-B-BL-083321 to 083322 • 1♂; Yunnan, Menghai County, Xishuangbanna Autonomous Prefecture; 1160 m alt.; 27–31.VI.1974; Yao Zhou & Feng Yuan leg.; SWU-B-BL-083323 • 1♂; Yunnan, Baihualing, Mt Gaoligong, Baoshan City; 1523 m alt.; 19.IV.2014; Yunkong Jiang & Tian Lu leg.; SWU-B-BL-083324 • 2♂; Yunnan, Hanlongzhai, Baihualing, Mt Gaoligong, Baoshan City; 1508 m alt.; 11.VI.2023; Xinran Li & Yifeng Liu leg.; SWU-B-BL-083325 to 083326 • 1♂; Yunnan, Yuxi City; 13.V.1980; Jingrong Zhao leg.; SWU-B-BL-083327.

#### Diagnosis.

Combining the following characteristics, this species is easily distinguished from other species of this genus: 1) pronotum with dense punctures; 2) the middle and hind femora with sparse spines; 3) body brown and cerci yellowish brown; 4) L4C with densely spiny process; 5) the end of L2 with one long spine; 6) the distal part of R1G with a thick spine; 7) the surface of the basivalvulae with furrows and microtrichia; 8) the end capsule of the spermatheca rod-shaped.

#### Description.

Sexual dimorphism present. ***Coloration*.** Male body brown to dark brown and female body black; ocelli white; cerci and styli yellowish brown (Fig. 1A–D).

**Male** (Fig. 1A, B). Body length including tegmen: 30.6–39.5 mm; body length: 20.7–29.8 mm; pronotum length × width: 4.3–6.8 mm × 6.6–9.2 mm; tegmina length × width: 26.0–32.9 mm × 7.4–10.2 mm. ***Head and thorax*.** Vertex slightly exposed. Interocular space slightly wider than the interocellar space, shorter than the distance between antennal sockets (Fig. 1E). Antennae longer than the body. Pronotum subelliptical, with the lateral edges not curved downward; anterior margin nearly concave, the median of hind margin convex; the widest point after the midpoint, the surface thin with dense punctures (Fig. 1F). The posterior-lateral angles of metanotum without projections (Fig. 1H). Tegmina and wings well developed, surpassing the tip of abdomen (Fig. 1A, B, I, J). Tegmina with ScP strong, posterior branch of R not reaching the end of tegmina (Fig. 1I). Legs slender. Front femur of type A_2_ (Fig. 1G). Mid- and hind legs with sparsely spines on ventral margin. Hind metatarsus approximately equal to the remaining segments combined. Pulvilli present on 1–4 tarsal segments, claws symmetrical and unspecialized, arolium small (Fig. 7A). ***Abdomen*.** First tergite of male abdomen with visible gland, setose gland sparse and not obscured by metanotum (Fig. 1H). Supra-anal plate short, lateral margin shrunken inward; the middle part of hind margin concave at an obtuse angle. Paraprocts (pp.) long, strip-shaped. Cerci robust (Fig. 1K). Subgenital plate nearly square; the hind margin arcuate, and the middle slightly concave. Styli symmetrical and apically rounded (Fig. 1L). ***Genitalia*** (Fig. 1M). L1 composed of one elongate sclerite and membrane bearing pubescence. L4C thin ribbon-like, with a densely spiny process near basal inner margin. L2 irregular, the end with one long spine inward. L3 unciform and well sclerotized, the basal part bifurcated. The distal part of R1H broad, the inner margin bifurcated with two small spines. The distal part of R1G with a slightly curved and thick spine inward.

**Female** (Fig. 1C, D). Body length: 21.6–24.7 mm; pronotum length × width: 6.4–8.2 mm × 8.5–10.5 mm; tegmina length × width: 6.3–7.5 mm × 5.1–6.4 mm. ***Head and thorax*.** Interocular space slightly wider than the interocellar space and the distance between antennal sockets (Fig. 1D). Pronotum subelliptical, the widest point near hind margin; anterior margin and hind margin straight (Fig. 1C). Tegmina and hind wings reduced. Tegmina squamiform, reaching the first tergite of abdomen; the outer margin oblique (Fig. 1C). Hind wings small lobe-like. Hind metatarsus approximately equal to the remaining segments combined. Pulvilli present on 1–4 tarsal segments, claws symmetrical and unspecialized, arolium smaller than male (Fig. 7A). ***Abdomen*.** Hind margin of tergum X (TX) with median invagination, and with a membranous line inside; cerci thick and upturned (easily broken) (Fig. 2A, D). ***Genitalia*** (Fig. 1N). First valve (v.I.) well sclerotized. First valvifer (vlf.I) small. Second valve (v.II) with strip-like sclerite. Posterior lobes of valvifer II (p.l.) irregular, the outer margin slightly connected with laterosternite IX (ltst.IX). Laterosternite IX broad and irregular. The base of anterior arch (a.a.) extended downward, surface densely covered with microtrichia. Spermathecal plate (sp.pl.) small, connected to basivalvulae (bsv.) by membrane. Spermathecal opening (sp.o.) located at the base of basivalvulae. Spermatheca branched, the leading duct short, the branching duct relatively long, and the end capsule rod-shaped (Fig. 7E). Basivalvulae bulbous and flared, surface with furrows and microtrichia. Laterosternal shelf (ltst.sh.) symmetrical, the base with furrows.

#### Ootheca.

11.4 mm long, 5.6 mm wide, reddish brown. Overall long, ridge slightly broad with serrations (Fig. 2A, B).

#### Etymology.

The species epithet is from the Latin word “*punctatus*”, which is in reference to the dense punctures on the pronotum.

#### Natural history.

This species has been found in the wild not far from human habitats, on roadsides or in grassy areas (Fig. 2C, D).

#### Distribution.

China (Sichuan, Yunnan).

#### Remarks.

Stochastic chiral dimorphism was found in male genitalia of this species. The genitalia of some male samples are left–right mirrored in comparison with common arrangement of Blattinae (Fig. 3A–C). We carefully examined all male specimens, and this phenotype was found in samples from three localities: Mt Ailaoshan (normal genitalia in two samples and mirrored genitalia in three samples), Baihualing (normal genitalia in one sample and mirrored genitalia in one sample) and Mt Daheishan (normal genitalia in one sample and reversed genitalia in one sample). In addition, there are no significant differences between the two kinds of genitalia, so they should be the same species. This is the first discovery of intraspecific genital chirality in Blattodea.

### ﻿ Vittiblatta ferruginea

Taxon classificationAnimaliaBlattodeaBlattidae

Luo & Wang sp. nov.

A618F8FF-386B-5472-9EAA-7D7235ACCB15

https://zoobank.org/450A2E05-DDC9-4601-9B07-6129BF4C8C79

**Figure 4.**
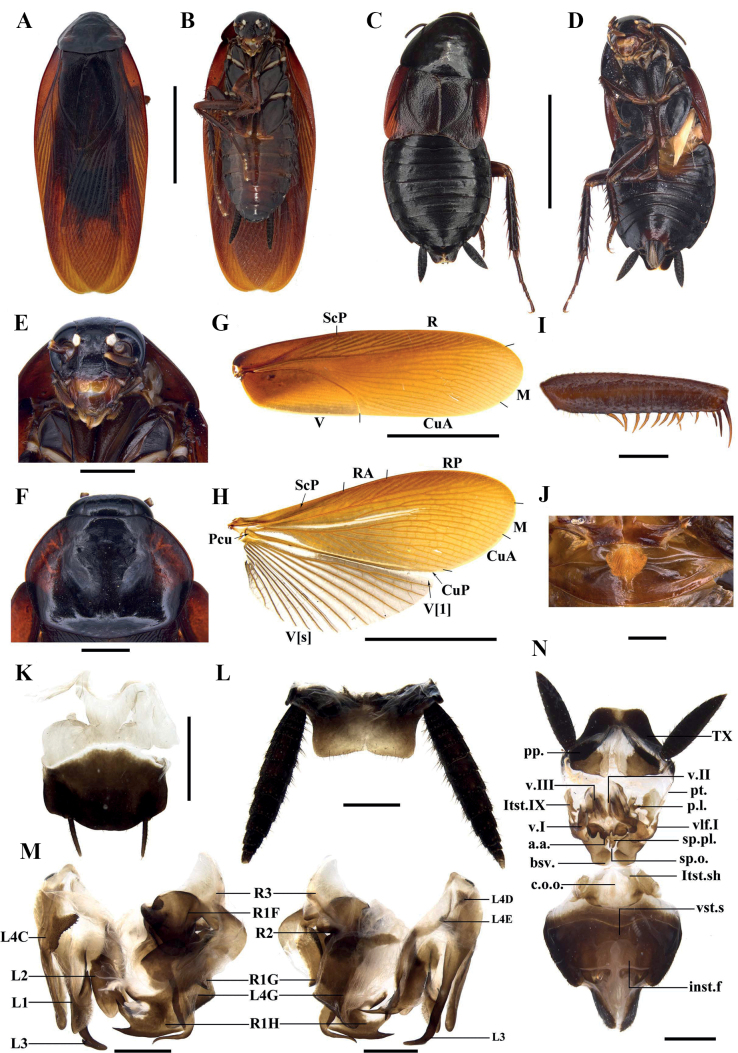
*Vittiblattaferruginea* Luo & Wang, sp. nov. **A, B, E–M** male holotype **C, D, N** female paratypes **A, C** habitus, dorsal view **B, D** habitus, ventral view **E** head **F** pronotum **G** tegmen **H** hind wing **I** front femur **J** hind margin of metanotum and tergal gland **K** subgenital plate, dorsal view **L** supra-anal plate, ventral view **M** male genitalia, dorsal (left) and ventral (right) view **N** female genitalia, dorsal view. Scale bars: 10.0 mm (**A–D, G, H**); 2.0 mm (**E, F, K, L, N**); 1.0 mm (**I, J, M**).

Figs 4 , 7 (in part)

#### Type materials.

***Holotype***: China • ♂; Yunnan, Tongbiguan Township, Yingjiang County, Dehong Dai and Jingpo Autonomous Prefecture; 1345 m alt.; 1.VI.2018; Lu Qiu & Wenbo Deng leg.; SWU-B-BL-082401. ***Paratypes***: China • 6♂♂; Yunnan, Tongbiguan Township, Yingjiang County, Dehong Dai and Jingpo Autonomous Prefecture; 1345 m alt.; 1–5.VI.2018; Lu Qiu & Wenbo Deng leg.; SWU-B-BL-082402 to 082407 • 6♂♂, 1♀; Yunnan, Meizihu Reservoir Highway, Pu’er City; 20–21.V.2016; Lu Qiu & Zhiwei Qiu leg.; SWU-B-BL-082408 to 082414 • 1♂; Xishuangbanna Tropical Botanical Garden, Chinese Academy of Sciences, Menglun Town, Mengla County, Xishuangbanna Prefecture; 27. V. 2016; Lu Qiu & Zhiwei Qiu leg.; SWU-B-BL-082415 • 1♂; Yunnan, Xiniu (Rhino) Plains Scenic Area, Pu’er National Park, Pu’er City, Pu’er National Park; 1602 m alt.; 2.VII.2021; Jiawei Zhang & Jinlin Liu leg.; SWU-B-BL-082416.

#### Diagnosis.

Combining the following characteristics, this species is easily distinguished from other species of this genus: 1) body dark reddish brown; 2) pronotum smooth without punctures; 3) the hind margin of subgenital plate arcuate; 4) the inner margin of L4C with serrate auriculate projection; 5) the distal part of R1G with a slender spine; 6) anterior arch with furrow; 7) the surface of basivalvulae flat.

#### Description.

Sexual dimorphism present. ***Coloration*.** Body reddish brown to dark reddish brown; ocelli white; cerci and styli brown to black (Fig. 4A–D).

**Male** (Fig. 4A, B). Body length including tegmen: 25.4–30.9 mm; body length: 18.2–21.9 mm; pronotum length × width: 4.6–5.9 mm × 5.8–7.0 mm; tegmina length × width: 22.5–26.9 mm × 6.3–8.2 mm. ***Head and thorax*.** Vertex slightly exposed. Interocular space slightly wider than the interocellar space, shorter than the distance between antennal sockets. Antennae longer than the body (Fig. 4E). Pronotum subelliptical; anterior margin slightly concave, hind margin slightly convex; the widest point near the midpoint (Fig. 4F). The posterior-lateral angles of metanotum with symmetrical and small projections (Fig. 4J). Tegmina and wings well developed, surpassing the tip of abdomen (Fig. 4A, B, G, H). Tegmina with ScP strong, posterior branch of R not reaching the end of tegmina (Fig. 4G). Front femur of type A_2_ (Fig. 4I). Mid- and hind legs with strong spines. Hind metatarsus longer than the remaining segments combined. Pulvilli present on 2–4 tarsal segments, claws symmetrical and unspecialized, arolium small (Fig. 7B). ***Abdomen*.** First tergite of male abdomen with visible gland, setose gland curved and downward (Fig. 4J). Supra-anal plate rectangular, lateral margin not shrunken inward; middle part of hind margin slightly concave. Paraprocts (pp.) long, strip-shaped. Cerci robust (Fig. 4L). Subgenital plate nearly square; the hind margin arcuate. Styli symmetrical and apically rounded (Fig. 4K). ***Genitalia*** (Fig. 4M). L1 composed of a elongate sclerite and membrane bearing pubescence. L4C thin, ribbon-like, the inner margin with serrate auriculate projection. L2 irregular, near distal part with two small spines and the end with one long spine inward. L3 unciform and well sclerotized, the basal part bifurcated. The distal part of R1H broad, the inner margin bifurcated with two small spines. The distal part of R1G with a curved, long spine inward.

**Female** (Fig. 4C, D). Body length: 22.2 mm; pronotum length × width: 6.1 mm × 7.4 mm; tegmina length × width: 5.8 mm × 5.2 mm. ***Head and thorax*.** Interocular space slightly wider than interocellar space and the distance between antennal sockets (Fig. 4D). Pronotum subelliptical, the widest point near hind margin; anterior margin and hind margin straight (Fig. 4C). Tegmina and hind wings reduced. Tegmina squamiform, reaching the first tergite of abdomen; the outer margin oblique (Fig. 4C). Hind wings small, lobe-like. Hind metatarsus approximately equal to the remaining segments combined. Pulvilli present on 2–4 tarsal segments, claws symmetrical and unspecialized, arolium smaller than male. ***Abdomen*.** Hind margin of tergum X (TX) with median invagination, and with a membranous line inside; cerci thick and not upturned (Fig. 4N). ***Genitalia*** (Fig. 4N). The base of first valve (v.I.) with dense microtrichia. First valvifer (vlf.I) thin. Second valve (v.II) with strip-like sclerite. Posterior lobes of valvifer II (p.l.) irregular, the outer margin disconnected with laterosternite IX (ltst.IX). Laterosternite IX broad and irregular. Anterior arch (a.a.) with furrow, and two symmetrical projections near outer margin, inner margin with microtrichia. Spermathecal plate (sp.pl.) broad, connected to basivalvulae (bsv.) by membrane. Spermathecal opening (sp.o.) located at the base of basivalvulae. Spermatheca branched, the leading duct short, the branching duct relatively long, and the end capsule unknown (Fig. 7E). Basivalvulae reniform, surface flat and margin with sparsely microtrichia. Laterosternal shelf (ltst.sh.) symmetrical.

#### Etymology.

The species epithet is from the Latin word “*ferrugineus*”, in reference to the reddish brown or dark reddish brown body.

#### Distribution.

China (Yunnan).

### ﻿ Vittiblatta undulata

Taxon classificationAnimaliaBlattodeaBlattidae

Luo & Wang sp. nov.

4F4420A0-BA54-5F0B-9A0C-7D9C8F97C1AD

https://zoobank.org/965CF6AF-9985-49EC-ADD6-15AFFB166498

**Figure 5.**
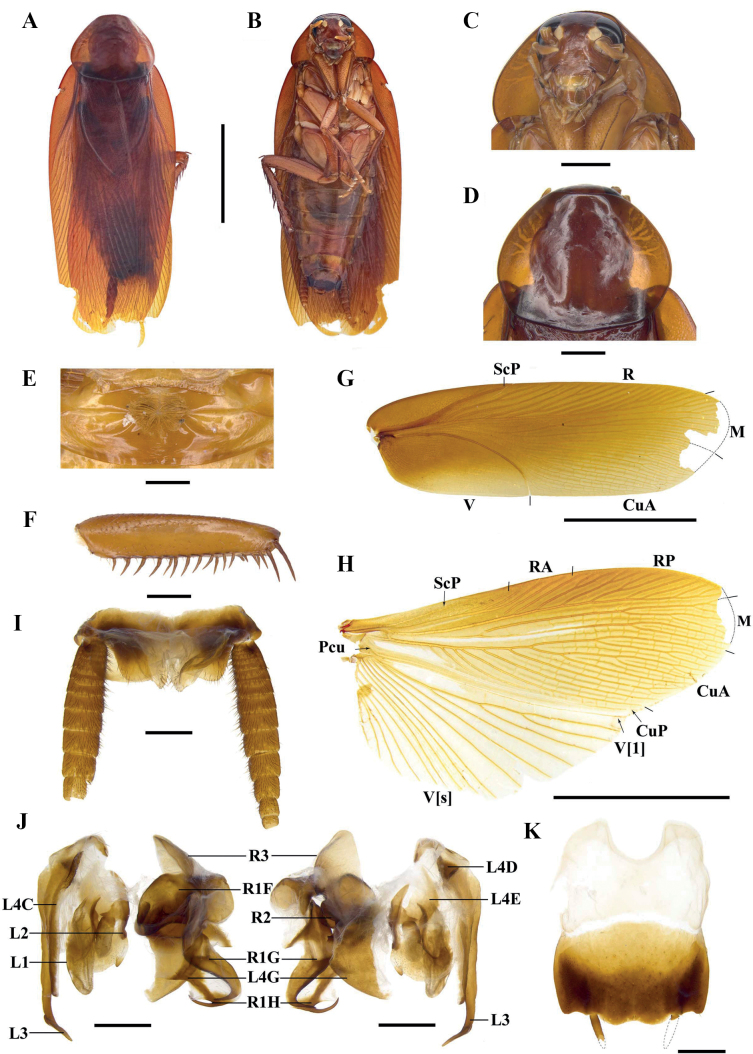
*Vittiblattaundulata* Luo & Wang, sp. nov. **A–K** male holotype **A** habitus, dorsal view **B** habitus, ventral view **C** head **D** pronotum **E** hind margin of metanotum and tergal gland **F** front femur **G** tegmen **H** hind wing **I** supra-anal plate, ventral view **J** male genitalia, dorsal (left) and ventral view (right) **K** subgenital plate, dorsal view. Scale bars: 10.0 mm (**A, B, G, H**); 2.0 mm (**C, D**); 1.0 mm (**E, F, I–K**).

Figs 5 , 7 (in part)

#### Type materials.

***Holotype***: China • ♂; Yunnan, Nabang Town, Yingjiang County, Dehong Dai and Jingpo Autonomous Prefecture; 282 m alt.; 11–13.VII.2012; collector unknown; SWU-B-BL-081901. ***Paratype***: China • 1♂; Yunnan, Nabang Town, Yingjiang County, Dehong Dai and Jingpo Autonomous Prefecture; 252 m alt.; 4.VI.2018; Lu Qiu & Wenbo Deng leg.; SWU-B-BL-081902.

#### Diagnosis.

Combining the following characteristics, this species is easily distinguished from other species of this genus: 1) body yellowish brown; 2) hind margin of subgenital plate wavy; 3) male genitalia L2 without spine at end, only a small protuberance; 4) the distal part of R1H broad, slightly sclerotized and hyaline, the end with an elongate and curved spine inward.

#### Description.

***Coloration*.** Body yellowish brown; ocelli white; hind margin of subgenital plate nearly brown (Fig. 5A, B).

**Male** (Fig. 5A, B). Body length including tegmen: 30.5–32.7 mm; body length: 27.1 mm; pronotum length × width: 6.2–6.6 mm × 7.7–8.2 mm; tegmina length × width: 24.9–25.4 mm × 8.0–8.2 mm. ***Head and thorax*.** Vertex unexposed. Interocular space slightly wider than the interocellar space, shorter than the distance between antennal sockets (Fig. 5C). Antennae longer than the body. Pronotum subelliptical; anterior margin straight, hind margin slightly convex; the widest point near the midpoint (Fig. 5D). The posterior-lateral angles of metanotum with symmetrical and small projections (Fig. 5E). Tegmina and wings well developed, surpassing the tip of abdomen (Fig. 5A, B, G, H). Tegmina with ScP strong, posterior branch of R not reaching the end of tegmina (Fig. 5G). Front femur of type A_2_ (Fig. 5F). Mid- and hind legs with strong spines. Hind metatarsus longer than the remaining segments combined. Pulvilli present on 1–4 tarsal segments, claws symmetrical and unspecialized, arolium small (Fig. 7C). ***Abdomen*.** First tergite of male abdomen with visible gland, setose gland curved, and directed toward left, right, and down (Fig. 5E). Supra-anal plate rectangular, lateral margin slightly shrunken inward; middle part of hind margin concave. Paraprocts (pp.) long, strip-shaped. Cerci robust (Fig. 5I). Subgenital plate nearly square; the hind margin wavy. Styli symmetrical and apically rounded (Fig. 5K). ***Genitalia*** (Fig. 5J). L1 membranous with pubescence. L4C thin and ribbon-like, the inner margin with a long projection of densely microtrichia. L2 irregular, the distal part with a small projection. L3 unciform and well sclerotized, the basal part bifurcated. The distal part of R1H broad, slightly sclerotized and hyaline, the end with an elongate and curved spine inward. The distal part of R1G with a curved, strong spine inward.

**Female.** Unknown, possibly brachypterous.

#### Etymology.

The species epithet is from the Latin word “*undulata*”, in reference to the way hind margin of subgenital plate.

#### Distribution.

China (Yunnan).

### ﻿ Planiblatta

Taxon classificationAnimaliaBlattodeaBlattidae

Luo & Wang gen. nov.

A56CCF60-EB1A-5330-AE85-435996002DB7

https://zoobank.org/35B1FFC2-904E-4A07-831B-637D0D94C776

#### Type species.

*Planiblattacrassispina* Luo & Wang, sp. nov.

#### Diagnosis.

This genus belongs to subfamily Blattinae (front femur of type A_2_, tarsi long and slender, cerci long and distinctly segmented, subgenital plate symmetrical) and can be distinguished from other genera of Blattinae by the following characters: 1) this sexually dimorphic genus can be distinguished from sexually monomorphic genera (see the diagnosis of *Vittiblatta* gen. nov.); 2) the genus (male macropterous, female micropterous) can be distinguished from the genera that female are apterous (see the diagnosis of *Vittiblatta* gen. nov.) and brachypterous (*Vittiblatta* gen. nov., *Cartoblatta*, *Scabinopsis*, *Bundoksia*); 3) hind metatarsus of this genus is longer than or equal to the remaining segments combined, distinguished from *Eumethana* and *Scabinopsis*; 4) visible tergal gland could be used to differ from *Cartoblatta*, *Periplaneta* s.s., and *Blatta*; 5) mesonotum and metanotum of this genus without finger-like projections can be distinguished from *Pseudoderopeltis*; 6) as mentioned in the diagnosis of *Vittiblatta* gen. nov., the difference of sclerite L4C and R1G within a genus is stable, this new genus (L4C curved and subhyaline, R1G with two curved, strong spines) can be distinguished from 10 genera of Blattinae (see the diagnosis of *Vittiblatta* gen. nov.).

#### Generic description.

Sexual dimorphism present. **Male.** Body flat. Antennae longer than the body. Pronotum subelliptical. The posterior-lateral angles of metanotum without finger-like projections. Tegmina and wings well developed, surpassing the tip of abdomen. Front femur of type A_2_; hind metatarsus equal to the remaining segments combined; pulvilli present, pulvilli of front metatarsus developed; claws symmetrical and unspecialized, arolium moderate. First tergite of the male abdomen with visible gland, setose gland not obscured by metanotum. Posterolateral corners of abdominal tergite V–VII produced. Supra-anal plate short, the hind margin slightly concave. The hind margin of subgenital plate straight. L2 folded, the dorsal sclerite broad, the distal part with one long spine. L3 unciform and the distal part bifurcated, longer than other sclerites. L4C less sclerotized, curved and subhyaline. The inner margin of R1H with two strong spines. The distal part of R1G with two curved and strong spines. **Female.** Tegmina and wings reduced. Tegmina small lobes. Hind wings absent. Spermatheca branched, the leading duct longer than the branching duct, the end capsule oval.

#### Etymology.

The generic epithet is from two Latin words “*plan*” and “*blatta*”, in reference to the flat male body. The gender of *Planiblatta* is feminine.

#### Distribution.

China (Yunnan).

### ﻿ Planiblatta crassispina

Taxon classificationAnimaliaBlattodeaBlattidae

Luo & Wang sp. nov.

A801B630-7467-5851-9D0E-4A20C3A6FDE3

https://zoobank.org/A47F1B88-7E4B-46D6-B9D4-FB3C9E6BE744

**Figure 6.**
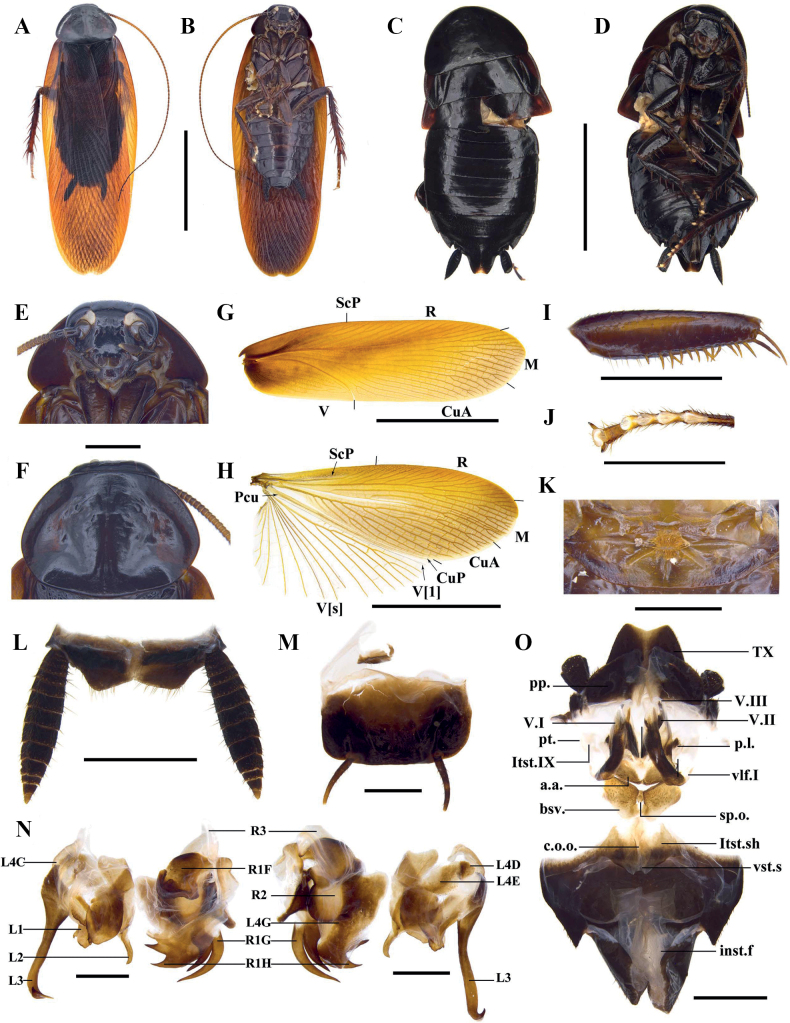
*Planiblattacrassispina* Luo & Wang, sp. nov. **A, B, E–K** male holotype **C, D, O** female paratypes **A, C** habitus, dorsal view **B, D** habitus, ventral view **E** head **F** pronotum **G** tegmen **H** hind wing **I** front femur **J** front tarsi **K** hind margin of metanotum and tergal gland **L** supra-anal plate, ventral view **M** subgenital plate, dorsal view **N** male genitalia, dorsal (left) and ventral (right) view **O** female genitalia, dorsal view. Scale bars: 10.0 mm (**A–D, G, H**); 2.0 mm (**E, F, I–L, O**); 1.0 mm (**M, N**).

**Figure 7.**
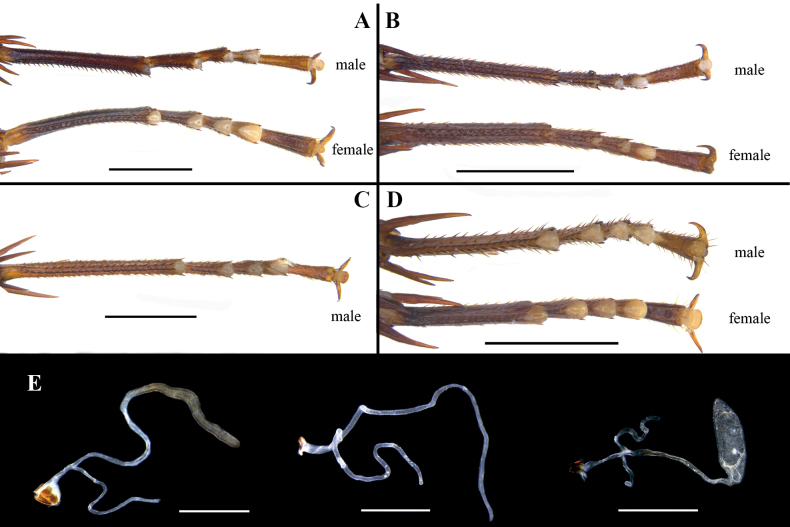
**A–D** hind tarsi **A***Vittiblattapunctata* Luo & Wang, sp. nov. **B***V.ferruginea* Luo & Wang, sp. nov. **C***V.undulata* Luo & Wang, sp. nov. **D***Planiblattacrassispina* Luo & Wang, sp. nov. **E** spermatheca, in order from left to right: *V.punctata* Luo & Wang, sp. nov., *V.ferruginea* Luo & Wang, sp. nov., *P.crassispina* Luo & Wang, sp. nov. Scale bars: 2.0 mm **(A–D)**; 0.5 mm **(E**).

Figs 6 , 7 (in part)

#### Type materials.

***Holotype***: China • ♂; Yunnan, Yaonan Village, Mt Ailaoshan, Xinping County, Yuxi City; 12.V.2016; Lu Qiu & Zhiwei Qiu leg.; SWU-B-BL-082901. ***Paratypes***: China • 3♂♂, 2♀♀; Yunnan; Yaonan Village, Mt Ailaoshan, Xinping County, Yuxi City; 11–12.V.2016; Lu Qiu & Zhiwei Qiu leg.; SWU-B-BL-082902 to 082906.

#### Diagnosis.

Combining the following characteristics, this species is easily distinguished from other Blattinae species: 1) pulvilli developed, pulvilli of front metatarsus occupy nearly 1/3 of ventral surface; 2) supra-anal plate short; 3) L4C curved and subhyaline, the base irregular; 4) the distal part of R1G with two curved and strong spines; 5) female tegmina small, lobe-like, and hind wings absent.

#### Description.

Sexual dimorphism present. ***Coloration*.** Body dark brown to black; vertex black; ocelli white; tegmina dark yellowish brown (Fig. 6A–D).

**Male** (Fig. 6A, B). Body length including tegmen: 28.5–30.1 mm; body length: 19.6–21.4 mm; pronotum length × width: 4.2–4.7 mm × 5.7–6.7 mm; tegmina length × width: 24–26.6 mm × 6.4–7.8 mm. ***Head and thorax*.** Vertex slightly exposed. Interocular space slightly wider than the interocellar space, slightly shorter than the distance between antennal sockets (Fig. 6E). Antennae longer than the body. Pronotum subelliptical; anterior margin straight, hind margin slightly convex; the widest point near the midpoint (Fig. 6F). The posterior-lateral angles of metanotum without finger-like projections (Fig. 6K). Tegmina and wings well developed, surpassing the tip of abdomen (Fig. 6 A, B, G, H). Tegmina with ScP strong, posterior branch of R not reaching the end of tegmina (Fig. 6G). Front femur of type A_2_ (Fig. 6I). Hind metatarsus equal to the remaining segments combined (Fig. 7D). Pulvilli present, pulvilli of front metatarsus developed, pulvilli of front metatarsus occupy nearly 1/3 of ventral surface (Fig. 6J). Claws symmetrical and unspecialized, arolium moderate (Fig. 7D). ***Abdomen*.** First tergite of male abdomen with visible gland, setose gland not obscured by metanotum and grown upward and downward (Fig. 6K). Posterolateral corners of abdominal tergite V–VII produced. Supra-anal plate short, lateral margin slightly shrunken inward; middle part of hind margin concave at an obtuse angle. Paraprocts (pp.) long, strip-shaped, the end curved downward. Cerci long and robust (Fig. 6L). Subgenital plate nearly square; the hind margin straight. Styli symmetrical and apically rounded (Fig. 6M). ***Genitalia*** (Fig. 6N). L1 membranous and irregular, margin thick. L4C curved and subhyaline, the base irregular. L2 irregular and folded, the dorsal sclerite broad, the distal part with a long spine. L3 unciform and well sclerotized, the basal part bifurcated. L4G strip-like. R1H slightly broad, inner margin of the distal part with two strong spines. The distal part of R1G with two curved, strong spines inward.

**Female** (Fig. 5C, D). Body length: 17.9; pronotum length × width: 5.4–5.6 mm × 7.9–8.0 mm; tegmina length × width: 3.5–3.7 mm × 2.3 mm. ***Head and thorax*.** Pronotum subelliptical, the widest point near hind margin; anterior margin curved, hind margin nearly straight (Fig. 6C). Tegmina and wings reduced. Tegmina small, lobe-like (Fig. 6C). Pulvilli present, pulvilli of front metatarsus developed, pulvilli of front metatarsus occupy nearly 1/3 of ventral surface (Fig. 6J). Claws symmetrical and unspecialized, arolium moderate (Fig. 7D). ***Abdomen*.** Hind margin of tergum X (TX) with median invagination, and with a membranous line inside (Fig. 6O). ***Genitalia*** (Fig. 6O). The surface of first valve (v.I.) with small punctures. First valvifer (vlf.I) slightly sclerotized and hyaline. Posterior lobes of valvifer II (p.l.) irregular, the outer margin unclear. Laterosternite IX (ltst.IX) slightly sclerotized and hyaline. Anterior arch (a.a.) with microtrichia near basal surface. Spermathecal plate (sp.pl.) nearly triangle. Spermathecal opening (sp.o.) located at the base of basivalvulae (bsv.). Spermatheca branched, the leading duct longer than the branching duct, and the branching duct also branched, the end capsule oval (Fig. 7E). Basivalvulae broad, surface with microtrichia; the left and right basivalvulae connected. Laterosternal shelf (ltst.sh.) symmetrical.

#### Etymology.

The species epithet is from the Latin word “*crassispinus*”, in reference to the two strong spines on the distal part of R1G.

#### Distribution.

China (Yunnan).

## ﻿Discussion

Deng et al. (2023) suggested that sclerite L4C has a high diversity in Blattinae, but we find this character, along with R1G, conservative in two new genera, and so in *Periplaneta* s.s. (Luo et al. 2023). We find that these two sclerites together can clearly distinguish *Vittiblatta* gen. nov. and *Planiblatta* gen. nov. from morphologically similar relatives, at least those examined in this study. Combined with external morphological characters, these two new genera are well supported. However, it needs to be confirmed whether L4C and R1G can be used to identify the genera not examined in this study, *Afrostylopyga*, *Apterisca*, *Cartoblatta*, *Brinckella*, *Deropeltis*, *Dorylaea*, *Eroblatta*, *Eumethana*, *Henicotyle*, *Macrostylopyga*, *Miostylopyga*, *Pseudoderopeltis*, *Scabinopsis*, and *Thyrsocera*.

Genital reversal within species is common in Blattodea, such as Blaberidae, most Pseudophyllodromiidae, and some Ectobiidae species (Brown 1975; Nieves and Bohn 1987; Klass 1997). However, our study is the first report of chiral dimorphism of male genitalia within a single species. Chiral dimorphism of male genitalia occurs rarely within an insect species, e.g. Coleoptera: Ahrens and Lago 2008, Hemiptera: Guglielmino et al. 2016, Lepidoptera: Nupponen 2009, Mantodea: Holwell and Herberstein 2010, Phasmatodea: Heleodoro 2022, Trichoptera: Botosaneanu and Hyslop 1998. Schilthuizen (2007, 2013) suggested that this phenomenon might be related to sexual selection. In *Drosophilamelanogaster*, this phenomenon is a result from mutations in the allele of Myo31DF (Hozumi et al. 2006; Spéder et al. 2006; Inaki et al. 2018), but whether it is the same in Blattodea needs to be investigated.

## Supplementary Material

XML Treatment for Vittiblatta

XML Treatment for Vittiblatta punctata

XML Treatment for Vittiblatta ferruginea

XML Treatment for Vittiblatta undulata

XML Treatment for Planiblatta

XML Treatment for Planiblatta crassispina

## ﻿Additional information

### Author ORCIDs

Xin-Xing Luo https://orcid.org/0009-0000-6838-1696

Wen-Bo Deng https://orcid.org/0000-0001-5796-241X

Yan-Li Che https://orcid.org/0000-0003-3214-9494

Zong-Qing Wang https://orcid.org/0000-0001-9413-1105

### Data availability

All of the data that support the findings of this study are available in the main text.
